# Spatio-Temporal Identification of Areas Suitable for West Nile Disease in the Mediterranean Basin and Central Europe

**DOI:** 10.1371/journal.pone.0146024

**Published:** 2015-12-30

**Authors:** Annamaria Conte, Luca Candeloro, Carla Ippoliti, Federica Monaco, Fabrizio De Massis, Rossana Bruno, Daria Di Sabatino, Maria Luisa Danzetta, Abdennasser Benjelloun, Bouchra Belkadi, Mehdi El Harrak, Silvia Declich, Caterina Rizzo, Salah Hammami, Thameur Ben Hassine, Paolo Calistri, Giovanni Savini

**Affiliations:** 1 Istituto Zooprofilattico Sperimentale dell’Abruzzo e del Molise ‘G. Caporale’, Teramo, Italy; 2 Société de Produits Biologiques et Pharmaceutiques vétérinaires (Biopharma), Rabat, Morocco; 3 Laboratory of Microbiology and Molecular Biology, University Mohamed V, Faculty of Science, Rabat, Morocco; 4 Istituto Superiore di Sanità, Reparto Epidemiologia delle Malattie Infettive, Centro Nazionale di Epidemiologia, Sorveglianza e Promozione della Salute, Rome, Italy; 5 Ecole Nationale de Médecine Vétérinaire de Sidi Thabet (ENMV), Sidi Thabet, Tunisia; University of Thessaly, GREECE

## Abstract

West Nile virus (WNV) is a mosquito-transmitted *Flavivirus* belonging to the Japanese encephalitis antigenic complex of the *Flaviviridae* family. Its spread in the Mediterranean basin and the Balkans poses a significant risk to human health and forces public health officials to constantly monitor the virus transmission to ensure prompt application of preventive measures. In this context, predictive tools indicating the areas and periods at major risk of WNV transmission are of paramount importance. Spatial analysis approaches, which use environmental and climatic variables to find suitable habitats for WNV spread, can enhance predictive techniques. Using the Mahalanobis Distance statistic, areas ecologically most suitable for sustaining WNV transmission were identified in the Mediterranean basin and Central Europe. About 270 human and equine clinical cases notified in Italy, Greece, Portugal, Morocco, and Tunisia, between 2008 and 2012, have been considered. The environmental variables included in the model were altitude, slope, night time Land Surface Temperature, Normalized Difference Vegetation Index, Enhanced Vegetation Index, and daily temperature range. Seasonality of mosquito population has been modelled and included in the analyses to produce monthly maps of suitable areas for West Nile Disease. Between May and July, the most suitable areas are located in Tunisia, Libya, Egypt, and North Cyprus. Summer/Autumn months, particularly between August and October, characterize the suitability in Italy, France, Spain, the Balkan countries, Morocco, North Tunisia, the Mediterranean coast of Africa, and the Middle East. The persistence of suitable conditions in December is confined to the coastal areas of Morocco, Tunisia, Libya, Egypt, and Israel.

## Introduction

West Nile Disease (WND) is a mosquito-borne disease caused by West Nile virus (WNV), a *Flavivirus* belonging to the Japanese encephalitis antigenic complex of the family *Flaviviridae* [1].

The WNV transmission cycle involves birds and mosquitoes. Wild and domestic species of birds act as amplifying and disseminating hosts while mosquitoes, particularly *Culex* spp., actively transmit the infection in bird populations. Humans, horses, and other mammals may incidentally be infected by a mosquito bite, yet they are not able to transmit the infection and are considered incidental or dead-end hosts [2,3]. Although frequently asymptomatic or characterized by mild febrile illness, in less than 1% of cases, infection in humans may develop severe neurological symptoms [4,5]. Clinical signs generally appear in 2–9 days in horses and in 2–14 days in humans [6–8].

WNV was isolated for the first time in a febrile patient from the West Nile district of Northern Uganda in 1937 [9]. From 1950 up to 1994, the virus was reported in different European, African, and Asian countries, but infection in humans, horses, and birds was mainly asymptomatic or mild [10–12]. Relevant clinical outbreaks were observed only in horses in Camargue (France) in 1962 and 1963 [13], and in humans in South Africa in 1974 [14].In the recent decades outbreaks of WND have been recorded in Eastern, Central, and Southern Europe [6,11,15–18], in Northern Africa [19–21], and in Israel from 1998 to 2000, where a significant mortality in birds was also observed [22]. Since 2008 WNV has spread into areas not previously affected, including Greece [23], Portugal [24], Turkey [25], and many eastern European countries (Albania, Bosnia, Bulgaria, Croatia, FYROM, Kosovo, Montenegro, Serbia) [26–28]. In the same period the disease has been reported in Israel, Italy, Spain, Hungary, Romania, Russia, and Ukraine [27–30].

The ecological aspects of WNV infection were first described in the 1950s in Egypt [31]. Since then, the relationship between the transmission cycle elements–birds (reservoir), mosquitoes (vector), equine and humans (dead-end hosts)–and the climatic and environmental factors have been extensively investigated.

The number of statistical and mathematical models aiming at exploring the association among the occurrence of WND and climatic and environmental variables, both in the Old and New World, has increased significantly over the past decade, and many factors have been proved to be of importance for WNV spread [32].

Temperature is one of the most important drivers in WNV transmission. Warmer air temperatures influence vector competence [33–35], by accelerating the virus replication within mosquito vectors and prolonging their breeding season [36]. Several studies showed a clear association between warmer temperature and outbreak intensity in Europe and USA [37–39]. Significant positive deviation of temperatures in Summer 2010 from perennial weekly average, calculated for the period 1981–2010, was proved to be associated with WND outbreaks in humans in Europe [37]. These results were further confirmed by Tran *et al*. [38], who demonstrated that anomalies of temperatures in July were the main predictors of WNV risk in Europe and neighbouring countries. Reisen *et al*. [40] indicate that the WNV spread into new areas from 2002 to 2004 in USA was associated with the occurrence of above-average Summer temperatures. Hahn *et*. *al* [39] found out that above average annual temperature was associated with national WNV disease incidence z-scores during 2004–2012 in the U.S.

The role of precipitation is controversial and scientific literature reports contradictory findings, as the timing and the amount of rainfall may have different effects on the density of adult mosquito population. The number of days with precipitation above the 95^th^ percentile value of monthly distribution or precipitation greater than 50 mm in a day, were proven to be positively correlated to the occurrence of WND in horses in Morocco and in humans in USA [41,42]. Whereas, positive correlation between WND cases and below-average precipitation has been found in Florida (USA) [43], which has been explained with a major concentration of nutrients for larvae in the remaining water pools. In addition, drought may facilitate a closer contact between birds and vector mosquitoes around the remaining water sources, thus facilitating the transmission of the virus [44]. The difference in the breeding habitat requirements for different *Culex* species can also help explain the contradictory effect of precipitation on WNV disease incidence [39]. Rainfall data mainly derive from ground measurements usually sparse and irregularly distributed on the territory; but other data sources, such as remote sensing data, offer an attractive alternative to ground measurements, enabling regular data collections, both spatially and temporally [45]. However, the coarse spatial resolution of these products (approximately 20 x 20 km) compared to the Mediterranean landscape, the ranges of available latitude, and the difficulty in validating these data, make vegetation indices more appealing as rainfall surrogate.

The Normalized difference vegetation index (NDVI) is the most common vegetation index used as covariate in WND risk studies [46,47]. It captures the strong relationship between vegetation cover and climatic elements, like temperature and rainfall, and can therefore be used as an indicator of climatic/environmental conditions suitable for vegetation growth and vector mosquito habitats. In a study of an outbreak of WNV encephalomyelitis in horses located in northern Indiana (USA), the median NDVI value for case premises within an identified cluster was significantly greater than the median NDVI calculated for other case and control premises [48]. Calistri *et al*. [41] investigated the possible association between NDVI and the occurrence of WND cases in 2003 and in 2010 in Morocco. The study noted that the NDVI values recorded during the Summers of 2003 and 2010, in the zones where WND occurred, were significantly higher than those registered during the same months in the rest of the decade.

Although the above variables are the ones more often investigated, many other predictors have been associated with WND occurrence. An extensive review of these factors can be found in two recent papers [32,47].

The different studies performed so far with the aim of establishing the association between the disease and possible risk factors, include various definitions of ‘case’, different predictors, and different geographical levels of aggregation (from geo-referenced cases to different administrative units–regions, province, district, etc.). This heterogeneity of approaches mainly derives from the complex epidemiology of WNV infection, which implies different proxies for WNV transmission [32]. It also depends on the different information collected by national surveillance plans [28]. The difficulty in collecting comparable data (in terms also of spatial location accuracy) and the complexity of the epidemiological relationships in WNV transmission cycle, force most of the studies to a national, regional, or local scale [49]. Although in U.S. and North America, since the last decade several studies have been conducted on a larger scale [42,50–52], in Europe studies focusing on a continental scale started to appear only during the last couple of years [37,38,53].

Without discounting the usefulness of the models already developed, the aim of this study is to analyse climatic and environmental data to identify, in space and time, areas potentially suitable for WND in Central Europe and in the Mediterranean basin.

## Materials and Methods

### Study area and data on WND cases

**Fig 1:**
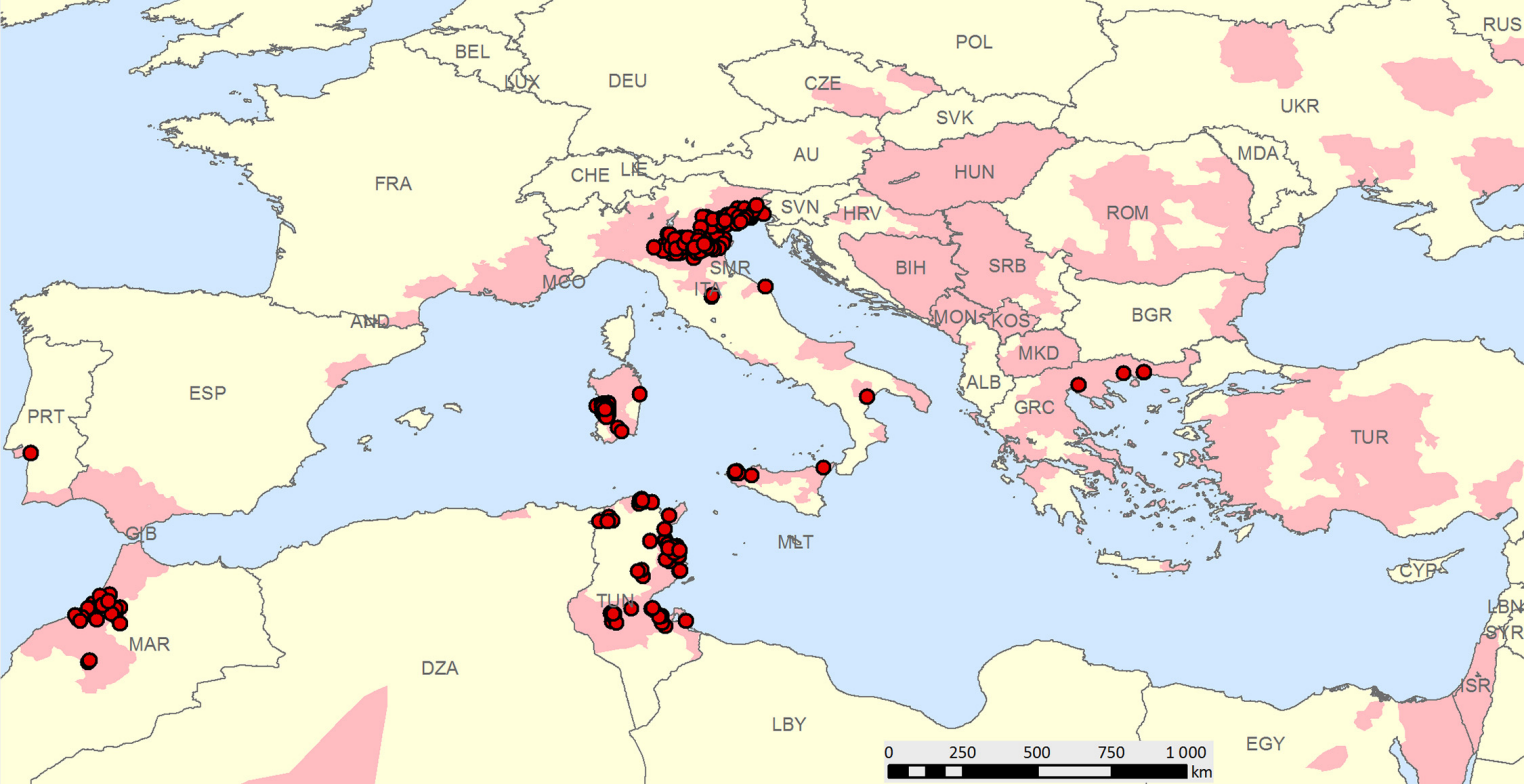
Study area: distribution of reported West Nile virus by country, region, and province since the 90s (light red polygons) and case locations included in the study between 2008–2012 (red points).

The study area was the Central Europe and the Mediterranean basin. It extended from 29.9° to 50°N and from 15.5° to 46.7°E and included 43 countries, 25 of which have experienced WNV in the recent decades. Autochthonous WND cases in horses (animals with clinical symptoms) and humans (West Nile neuroinvasive disease and West Nile fever syndromes) which occurred between 2008–2012 were included in the study. For each case, information about clinical signs onset date along with the address of infected patients or coordinates of the place where equine cases occurred were recorded into the database. A total of 274 case-locations were analysed (Fig 1). Horse cases revealed by serology only were not considered, due to the difficulty of tracing back the date of infection.

Equine clinical cases (4 cases for Greece and 1 for Portugal) were from OIE World Animal Health Information System (WAHIS) [24], from the Italian National Surveillance Information System (111 cases), and from the Moroccan National Laboratory BIOPHARMA (Société de Productions Biologiques et Pharmaceutiques Veterinaries, RABAT) (23 cases). Data on human cases were provided by the National Centre for Epidemiology, Surveillance and Health Promotion of the the Istituto Superiore di Sanità for Italy (52 cases) and by the Primary Health Care Directorate (DSSB) of Ministry of Health in Tunisia (83 cases). A minimal data set underlying the findings in the study is reported in S1 Table (Italian data on humans have been removed because of legal restriction).

Data on humans were collected from the National Surveillance Systems for communicable diseases in force in Tunisia and Italy. The use of data was approved by the Ministries of Health of the different Countries; all data on patients were treated anonymously and de-identified prior to analysis.

### Climatic and environmental data

All human and equine cases enrolled in the study have been assumed to be exposed to the infection in the place where the disease was detected and notified and, therefore, their geographical locations were used to extract the values of climatic and environmental variables.

The selection of predictors used to characterize places in which WND cases occurred was based on literature and raster data availability for the Mediterranean basin and Central Europe. Data were divided in two groups: static and dynamic. The static variables were altitude and slope, whereas the dynamic ones were the night time Land Surface Temperature (LSTN), the Normalized Difference Vegetation Index (NDVI), the Enhanced Vegetation Index (EVI), and the daily temperature range; the latter variable expressed as the absolute value of the difference between the LSTN and the daytime land surface temperature (LSTD).

Altitude values were extracted from the Global 30-Arc-Second Elevation Dataset for the World, developed by the United States Geological Survey (USGS) (http://eros.usgs.gov, last access on 03/21/2014). Slope data were derived using the Spatial Analyst extension in ArcGIS 10 software (ESRI® Inc., Redlands, CA).

NDVI and EVI were extracted from MOD13Q1 NASA product (250 m spatial resolution and a temporal resolution of 16 days) and LSTN and LSTD were extracted from MOD11A2 NASA product (1 km spatial resolution, temporal resolution 8 days) for the 2008–2012 period. Data were downloaded from the Land Processes Distributed Active Archive Center (LP DAAC) service at NASA website, (http://e4ftl01.cr.usgs.gov/MOLT, accessed on the 06/10/2014). Data were first submitted to a pre-processing phase of missing data interpolation to fill in pixels without data. All the data were then aggregated (average) at the same temporal resolution of 16 days.

All the environmental and climatic datasets were converted to Geotiff raster format with the same extent, spatial resolution (5 km), and Geographic Reference System (GCS- WGS84).

To cover the entire extent of the Mediterranean basin, 1,840 images were downloaded and processed for LST (46 images per year, 5 years, 8 tiles) and 920 for NDVI and EVI (23 images per year, 5 years, 8 tiles).

### Statistical analysis

#### Analysis steps

The analysis was based on two main steps: the first (Mahalanobis Distance analysis) aimed at identifying geographical areas ecologically similar to those where equine and human WND cases were detected; the second one (potential mosquito growth) aimed at monitoring the daily growth rate of mosquitoes, based on environmental temperature. The two outputs were then combined to take into consideration both the environmental suitability and the seasonality of mosquito growth during the year. The analyses were run using ArcGIS 10 (ESRI® Inc., Redlands, CA) and R software, version 2.13.1 (R Development Core Team, 2011).

#### Mahalanobis analysis

The first step was to associate each WND case to the corresponding value of each predictor. In particular, each WND case was assigned to one of 118 16-days periods (from the 1^st^ of January 2008 to the 29^th^ of December 2012), according to the date of clinical symptoms onset. Then, the value of each dynamic predictor (NDVI, EVI, LSTD, LSTN), calculated in the same 16-days period, was associated to the WND case.

The Mahalanobis Distance Statistic (MD) [54] was calculated for the entire set of raster predictors per each year (from 2008 to 2012).

The Mahalanobis distance in each pixel was calculated as follows:

$$MD={\left(X-m\right)}^{T}C^{-1}\left(X-m\right)$$

where *X* is the vector of environmental and climatic data for each pixel in the raster, *m* is the vector of mean values of independent variables for the areas location with WND human and equine cases, *C* ^-1^ is the inverse covariance matrix of independent variables for the areas with WND human cases, and *T* indicates a vector should be transposed.

The MD statistics produced 23 MD values for each pixel in each year. To be able to combine MD results and daily growth rate of mosquitoes, the MD values for the same 16-days period in the different years were interpolated through a cubic spline function (‘splinefun’ in R package ‘stats’) returning 365 MD values for a hypothetic year. S1 Fig shows two examples of the 23 MD values obtained each year from 2008 to 2012, and the spline interpolation used to test the significance of the MD values.

The estimated daily MD statistics follows an approximate Chi-square distribution with n-1 degrees of freedom, where n is the number of predictors. The analysis produced 365 raster images, in which for each pixel the p-value was reported. P-values close to zero indicate that the climatic and environmental conditions are statistically different from those present in pixels where WND cases were present.

#### Mosquito growth

The mosquito lifecycle was modelled considering two age compartments model: an aquatic stage (eggs, larvae, pupae) called “L”, and a terrestrial stage (adult mosquitoes) with “N” being the total density of adult mosquitoes [55]. The model, driven by environmental temperature (T), was implemented considering density-dependent population growth rates of mosquitoes (bounded by the carrying capacity of the mosquito larvae), daily temperature, and daytime length at every geographical latitude. The following system of Ordinary Differential equations (ODEs) was implemented:

$$\{\begin{array}{l} \frac{dL}{dt}=\left(b_{L}\left(T\right)\delta \;N-m_{L}\left(T\right)L\right)\left(1-\frac{L}{K}\right)-b_{N}\left(T\right)L\\ \;\frac{dN}{dt}=b_{N}\left(T\right)L\;-m_{N}\left(T\right)N \end{array}$$

The birth rate functions for larvae (*b*_*L*_) and adults (*b*_*N*_), the fraction of active mosquitoes *δ*, the mortality rate for adults *m*_*N*_(*T*) were derived from Rubel *et al*. [55], all functions are described in Table 1. The mortality rate for larvae (*m*_*L*_) was modeled through a U-shape function according to Beck-Johnson *et al*. [56] in order to consider a wider range of temperature activity, and lower mortality rate at higher temperatures. Enlarging the range of temperatures with mosquito activity was necessary to adapt a model developed at Austria latitudes to all Mediterranean locations. Table 1 shows detailed formulas, parameters, and references of the mosquito growth model. Birth and mortality parameters and initial values adopted in the ODEs are reported in S2 Table.

**Table 1:** Formulas, parameters, and references of the mosquito growth model: ordinary differential equations.

Parameter	Value	Description	Reference
**L**	L(temperature, latitude, day of the year)	Density function of larvae	
**N**	N(temperature, latitude, day of the year)	Density function of adult mosquitoes	
**T**	degree Celsius	Temperature in degree Celsius	
***b***_***L***_	*b*_*L*_(*T*) = 2.325 *k*(T)	birth rate of larvae	[55]
***b***_***N***_	*b*_*N*_(*T*) = *b*_*L*_(*T*)/10	birth rate of adult mosquitoes. It is of the same shape as larvae birth rate, one order of magnitude lower.	[55]
**K**	3,300,000	carrying capacity, bounding the growth of mosquito larvae population	[57]
***m***_***L***_	$m_{L}\left(T\right)\;=\;\gamma_{L}\;\mu_{3}\;exp\left({\left(\frac{T-\mu_{4}}{\mu_{5}}\right)}^{2}\right)$	Mortality rate of larvae. It is an U-shaped function.	[56]
***m***_***N***_	*m*_*N*_(*T*) = *m*_*L*_(*T*)/10	Mortality rate of adult mosquitoes. It is of the same shape as larvae mortality rate, one order of magnitude lower.	[55,56]

The ODEs were run considering daily temperature data calculated as follow: for each 16-days period of each year (from 2008 to 2012), the mean daily temperature was calculated for each pixel, averaging LSTN and LSTD. This calculation produced 23 mean temperature values per each pixel and each year. The values of the same 16-days period in the different years were interpolated through a cubic spline function (‘splinefun’ in R package ‘stats’) to obtain a series of 365 values (referred to as a hypothetic year).

The outputs were represented in 365 raster images with the modelled number of adult mosquitoes for each pixel derived from an initial value of 500,000.

To integrate mosquito growth values with the outputs of the Mahalanobis analysis, the estimated numbers of adult mosquitoes were normalized in a [0–1] interval, by dividing each value in each pixel by the maximum value reached in the pixel. This permitted to include the seasonal pattern of mosquito population, without having to take into account the absolute values of abundance (that is unknown), but considering the probability for the mosquito population of being at its maximum in a pixel in a day.

#### Mapping suitable areas for WND

**Fig 2:**
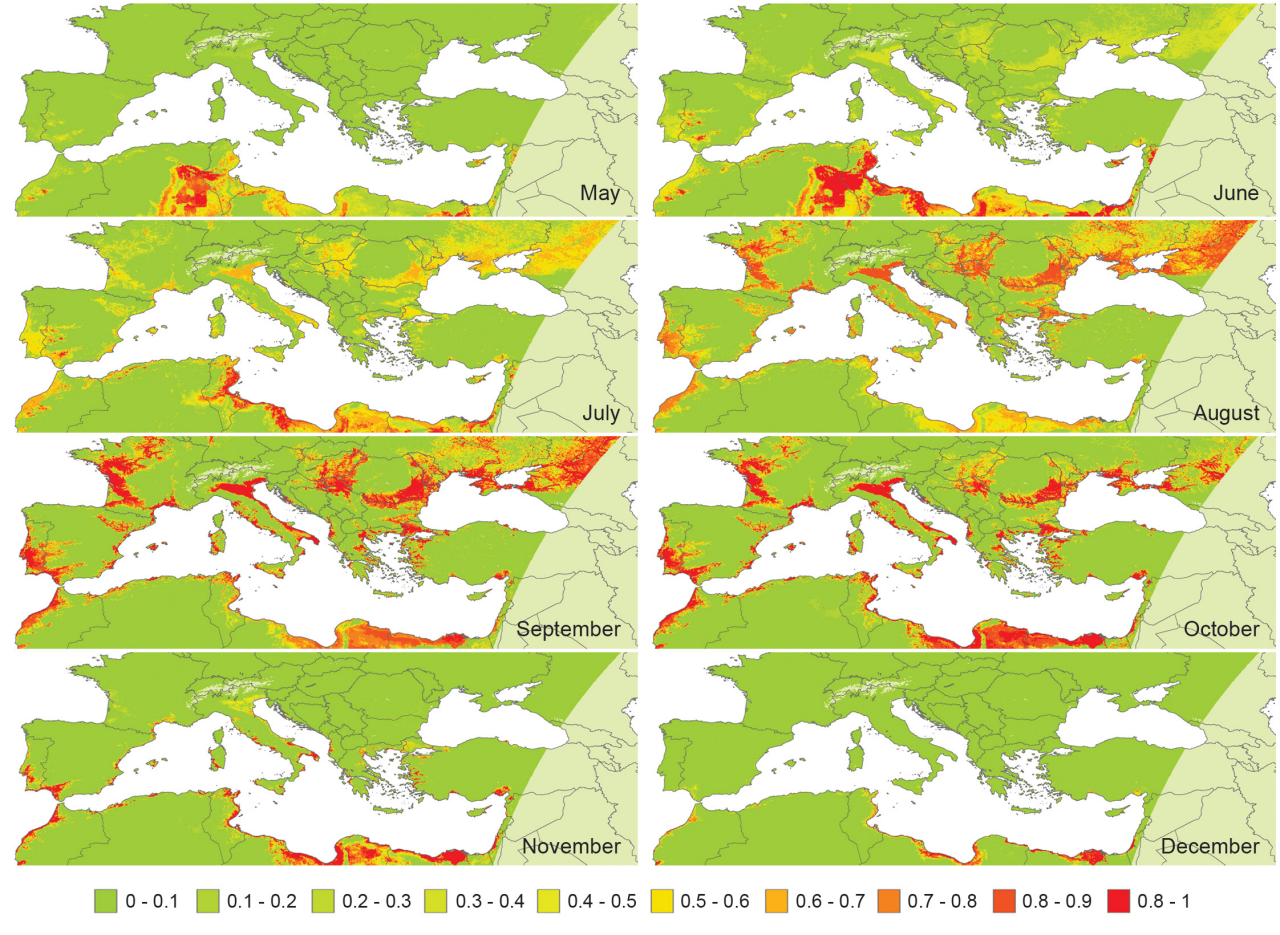
West Nile Disease suitability maps (on a monthly basis) from May to December for the study area. From January to April, no areas suitable for WND were predicted (probability was less than 1%).

The 365 raster images of MD p-values and the 365 raster images of normalized mosquito growth values were then combined with an ‘AND’ operator to produce 365 maps of suitable areas. Finally, the 365 results were aggregated considering the maximum value in a 30-day period producing 12 suitability maps (Fig 2).

#### Validation

The suitability maps were validated using the receiver operating characteristic (ROC) curve method [58]. The ROC area under the curve (AUC) assesses the capacity of the model to discriminate the locations where the WND is present from those where it is absent. AUC values can be interpreted on the scale proposed by Swets [59]: AUC<0.7 poor; 0.7 < AUC < 0.90 useful; AUC > 0.9 good. The method requires independent data representing both disease presence and absence.

Considering the limited number of cases, which made impossible a division into training and testing sets, k-fold cross validation was used. The original dataset on WND occurrence was randomly partitioned into k subsamples of equal size. Of the k subsamples, one subsample was used for the model validation, and the other k-1 subsamples were used as model’s input data. The cross-validation process was then repeated k times, for each of the k subsamples. In particular, the 274 cases, were randomly subdivided into k partitions, with k = 7. In each of the 7 iterations, 234 cases were used as input data and 40 for the validation of the model (in the last iteration 240 data points were used as model’s input and 34 for the validation). As far as negatives are concerned, 600 background data points for each iteration were created. These data were randomly generated through a selection of 50 points in each of the 12 final prediction results (to take into account the different predictions in the different months). The random selection occurred within a defined area based on the following criteria: (i) more than 5 km away from a disease occurrence point (*i*.*e*. occurrence and background points could not occur in the same cell), (ii) more than 5 km away from another background point, to ensure that there was no more than one background point per cell, (iii) the points were randomly located on land.

In each iteration the AUC was calculated and the mean value of the 7 AUC derived from the k-fold cross validation was retained.

## Results

### Suitability maps

The final 12 suitability maps, derived by the combination of MD analysis and the mosquito growth model, revealed an interesting spatio-temporal pattern of WND distribution between May and December (Fig 2).

During the period from May to June, the more suitable areas are recorded in Tunisia (eastern coast and central part), Libya, Egypt, and Northern Cyprus; while suitable conditions start to be recognised also in the European continent only in July. The following months (August, September, and October) show increased significance in Italy, France, Spain, the Balkan countries, Morocco, northern Tunisia, and all along the Mediterranean coast of Africa and Middle East. In November, Europe returns to unsuitable conditions, with the exception of limited coastal areas facing the Mediterranean Sea (Italy, France, Spain, and Greece). The persistence of suitable conditions in December is confined to coastal areas in Morocco, Tunisia, Libya, Egypt, and Israel.

The contribution of the mosquito growth model was significant in the final prediction, accounting for extreme temperatures (either too high or too low) unsuitable for mosquito survival. An example of seasonality is reported in S2 Fig. The graph shows how the potential availability of mosquitoes varies in three different points: desert in Algeria, Padan Plain, and Apennine Chain (at an altitude of 1000 m). In the desert, temperature was suitable for mosquito survival only between April and June, whereas in temperate regions such as Padan Plain, the survival period is longer and lasts from May to November.

### Validation

**Fig 3:**
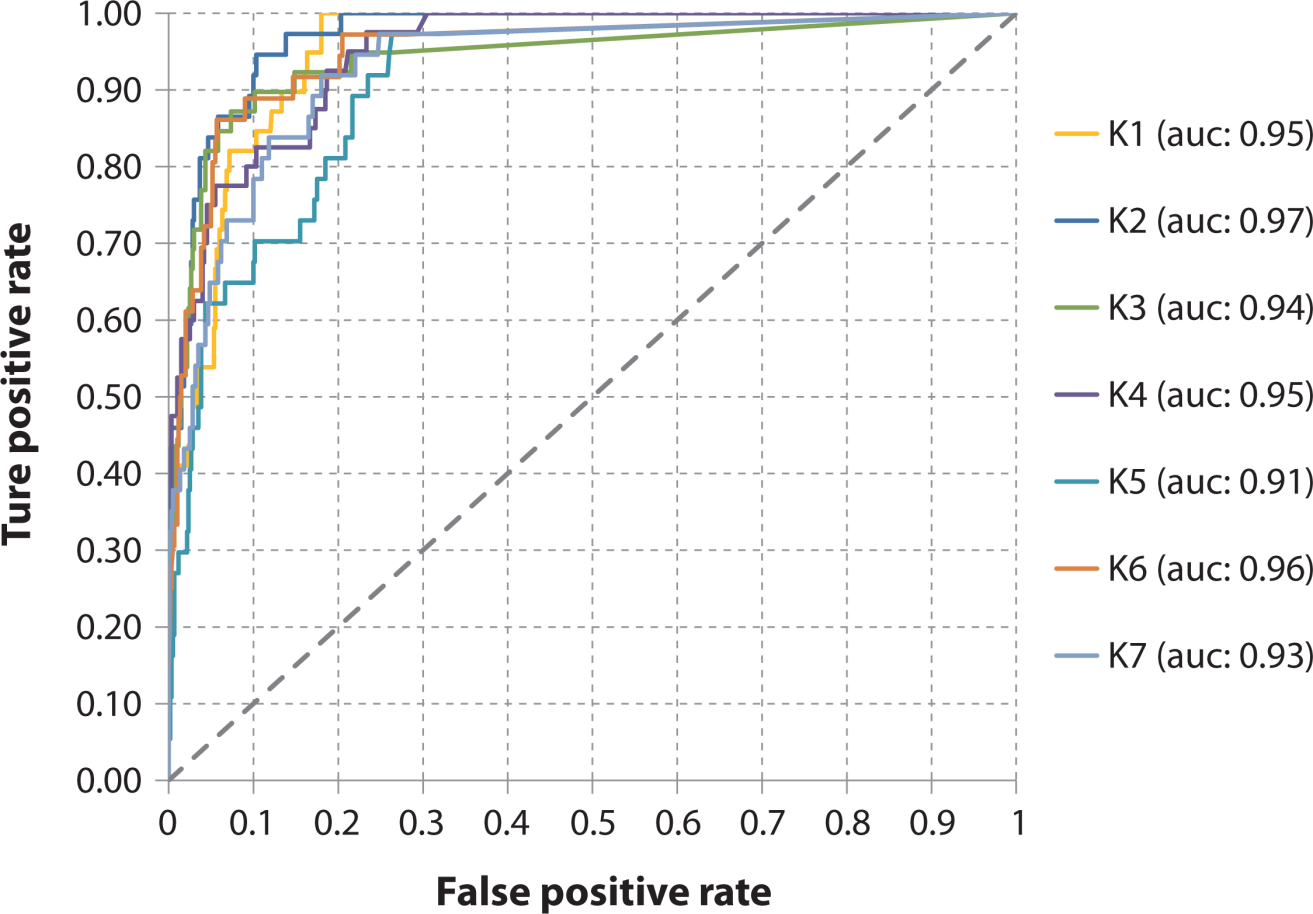
Validation of West Nile Disease presence prediction: receiver operating characteristic (ROC) curves in the k-fold (k = 7) iterations.

According to the ROC method, the capacity of the model to discriminate between presence/absence locations was excellent: all the iterations had an AUC higher than 0.90, with an average value of 0.944 (ranging from 0.91 to 0.97) (Fig 3).

## Discussion

Both biotic and abiotic factors contribute to the WND occurrence and spread. These factors include: favourable climatic conditions, suitable habitats for mosquito reproduction and survival, the presence of migratory birds settlements, the existence of local bird populations able to sustain the WNV transmission and amplification, the presence of competent bridge vectors for the transmission to humans, and the presence of susceptible human and equine populations [32,47,51]. In this study, a two-step approach was used to identify suitable areas for WND in Central Europe and Mediterranean basin. In a first step, the MD statistic was applied to climatic and environmental variables, identifying areas similar to those where WND has occurred in the past. The MD model was based on the geographical location of WND horse and human cases reported in the study area from 2008 to 2012. These data are considered more reliable than records on WNV detected in mosquitoes or birds, for which the geographical dispersal range would have been difficult to estimate [60,61]. In a second step, the normalized abundance of mosquito population across the year was estimated through a compartment model [55,57], considering daily temperature and daytime length at each geographical latitude. The temporal component (included in both the MD and mosquito growth models) permitted to combine the two steps and contributed to identifying risk areas in space and time. The obtained results are consistent to the WNV spread recorded in Europe and in the Mediterranean basin and are in accordance to similar studies performed locally [49]; potential new areas at risk are also identified providing public health authorities with information on targeted areas to implement preventive/surveillance measures.

In Northern Africa the model identified areas where WNV actually circulated in the past: the Egyptian coast [62], Tunisia [63], and Morocco [20,41]. In the eastern part of the Mediterranean basin, Israel represents a country with a long experience of WNV circulation since the ‘50s [64], as well as an important point of entry for pathogens, including those carried by migratory birds flying from Asia. Phylogenetic studies identified the strain circulating in Israel in 1998 as the likely ancestor of the virus introduced into North America the following year [65,66]. A recent review on WNV infection in Israel indicates that nearly 1,400 human cases have been reported from 2000 to 2012, with the highest incidence in the coastal cities and peaks in the late Summer months and early Autumn [67]. These findings are fully in agreement with the spatio-temporal distribution of the model estimates.

In the Balkans, suitable areas were predicted in Romania, with higher risk values in the proximity of the Danube delta. Since 1996, Romania experienced several WND cases, both in humans and in horses [15,68–70]. In 2010 human cases were confirmed in the central and northern provinces of Romania [71], zones correctly identified at major risk by the model. Other areas in Croatia, Serbia, Bosnia-Herzegovina, and in Russia were identified as suitable. In particular, in Russia, the model indicated the provinces of Astrakhan, Volgograd, and Rostov as at major risk. Actually, these provinces represent the “core” of WNV circulation in Russia, accounting for the 58% of the 2,283 human cases recorder in the country from 1999 to 2013 [72].

In Southern Europe, the Camargue region in France was confirmed to be a suitable area. This region experienced WND several times in the past, both in humans and horses [73–75]. More recently (from 2003 to 2006), neurological syndromes in horses were observed also in the Var and Eastern Pyrenees Departments [71].

The model predicted high levels of suitability in the western inland part of France, where WNV infection has not been detected. Though all the considered climatic and environmental variables tend to support the possibility of occurrence of mosquito-borne infections, the reasons for the absence of WNV circulation in this area of France are difficult to be determined. A possible explanation could be the absence of wetland locations used by migratory birds as resting sites [38]. Western migratory flyways only marginally touch the area, being confined along the Atlantic coast [38].

In Spain, human WND cases were reported in the 60s and the 70s in the northwest of the country and in the Ebro delta region, respectively [76]. The Ebro delta is a stopping-off point for birds migrating between African and European regions. Recently the disease was reported in Spain in both humans (2001, 2004) and horses (2007–2008). In September 2010 the first clinical case of WND was detected in a horse in Cadiz, the southernmost province of Spain, and 35 further cases were reported in three provinces of Andalusia, in southern Spain [77] up to December 2010. Between September and October 2011, five new cases in horse stables were confirmed in the same area [71,78]. Concordantly, the model predicts high probability of disease occurrence in southern areas of Spain, close to Portugal border, all along the Mediterranean coast and the Ebro valley from August till November.

In Greece the model has produced results comparable to those obtained by Valiakos *et al*. [49], who used wild bird surveillance and human data collected between 2010 and 2012 to identify areas at risk for WND. They identified clusters of disease and potential risk areas in places at low altitudes and in proximity of water, thus identifying wetland habitat used as resting and breeding sites for migratory and resident birds.

One of the areas underestimated by the model was Turkey. In the last decade, WNV circulation in Turkey has been extensively documented in humans, birds, mosquitoes, and horses [25,79–81]. The model identified the Mediterranean coastal zones of Turkey as suitable for WNV circulation, but underestimated the risk in other areas, such as the Central Anatolia, where the infection was reported in the past in horses and humans [25,79]. Other climatic and environmental factors, such as marshy areas and resting sites for migratory birds, already identified as relevant in Greece [82] as well as in Tunisia [63], might play a significant role and a more accurate investigation is needed.

The model identified suitable areas in countries that have never reported the disease (e.g. Libya). These areas deserve a deeper analysis to understand if the absence of the disease in the countries is associated to a lack of surveillance, to underreporting, or if other climatic and environmental characteristics might inhibit the spread of the disease.

At the same time a possible overestimation by the model cannot be excluded. In Europe, transmission of WNV has two basic cycles and ecosystems: rural (sylvatic) cycle and an urban cycle [83,84]. Biotic and abiotic factors characterizing the two cycle can be significantly different (e.g. altitude, slope, vegetation cover). In our study, clinical cases associated to both cycles were included in the analysis, this might have brought a greater variability for some climatic and environmental variables with a potential overestimation of some areas in terms of risk.

The two most relevant variables in driving the MD results were altitude and slope. This was derived analyzing the box-plots of predictors in two groups: in pixels with high similarity (MD p-value> = 0.75) and in pixels with low similarity (MD p-value < = 0.25). For all the predictors, the variability in the two groups was very similar, with the exception of altitude and slope, in which dispersion in the high similarity pixels was much lower than the dispersion in the low similarity pixels.

The limited number of cases for which the exact geographical location was known seems to have not affected the accuracy of the outcome, confirming areas in which WND occurred in the past, but also revealing new risk areas, which require further investigation. Nevertheless, the accuracy of the model could be improved by adding additional high resolution geolocated WND cases, as the information on time and place of exposure become available. Another component certainly to be improved is the estimate of the seasonal abundance of vectors, this could be done considering additional variables, such as the presence of water, important for the availability of mosquito breeding sites [38] or acquiring longitudinal entomological data. This can be achieved, though, only through a supranational collaborative approach, needed to better understand the mechanisms behind WNV dissemination and to effectively monitor the spread of the infection. International surveillance systems, based on agreed standardised criteria [85] and able to function as effective early warning systems along with the availability of accurate disease data are pivotal in preventing the spreading of infectious diseases [86]. Spatial models based on MD analysis can be successfully applied to several vector borne diseases, in which climatic and environmental characteristics play a fundamental role (i.e. Bluetongue, African horse sickness, Rift Valley fever, Crimean Congo haemorragic fever, etc.).

The suitability maps produced in this study provide a valuable tool for better planning surveillance activities and fine-tuning preventive measures to decrease the risk for WND occurrence in both humans and animals.

## Supporting Information

S1 Fig Examples of the 23 MD values in each of the studied year and the spline interpolation in one point in Northern Italy (10.9863E, 45.4293N)—A- and one point in the desert of Algeria (1E, 31N)- B. The different scales are meant to highlight the differences in the MD values.
(EPS)

S2 Fig Standardized mosquito abundance across a year in three different points: desert in Algeria, Padan Plain and Apennine Chain (at an altitude of 1000 m). (JPG)

S1 Table Minimal data set underlying the findings in the study. Data include equine clinical cases in Italy, Morocco Portugal and Greece; human cases in Tunisia.
(XLSX)

S2 Table Formulas, parameters, initial values and references of the mosquito’s growth model: birth and mortality parameters in the ordinary differential equations (ODEs). (DOCX)

## References

[1] Karabatsos N. International catalogue of arboviruses, including certain other viruses of vertebrates 3rd ed. and Supplements 1986–98. San Antonio TX Am Soc Trop Med Hyg 1985; 10.4269/ajtmh.1978.27.372 646031

[2] Kulasekera VL, Kramer L, Nasci RS, Mostashari F, Cherry B, Trock SC, et al West Nile virus infection in mosquitoes, birds, horses, and humans, Staten Island, New York, 2000. Emerg Infect Dis. 2001;7: 722–725. 10.3201/eid0704.010421 11589172 PMC2631749

[3] Bunning ML, Bowen RA, Cropp B, Sullivan K, Davis B, Komar N, et al Experimental infection of horses with West Nile virus and their potential to infect mosquitoes and serve as amplifying hosts. Ann N Y Acad Sci. 2001;951: 338–339. 11797793 10.1111/j.1749-6632.2001.tb02712.x

[4] Mostashari F, Bunning ML, Kitsutani PT, Singer DA, Nash D, Cooper MJ, et al Epidemic West Nile encephalitis, New York, 1999: results of a household-based seroepidemiological survey. Lancet Lond Engl. 2001;358: 261–264. 10.1016/S0140-6736(01)05480-0 11498211

[5] Campbell GL, Marfin AA, Lanciotti RS, Gubler DJ. West Nile virus. Lancet Infect Dis. 2002;2: 519–529. 12206968 10.1016/s1473-3099(02)00368-7

[6] Cantile C, Di Guardo G, Eleni C, Arispici M. Clinical and neuropathological features of West Nile virus equine encephalomyelitis in Italy. Equine Vet J. 2000;32: 31–35. 10661382 10.2746/042516400777612080

[7] Bunning ML, Bowen RA, Cropp CB, Sullivan KG, Davis BS, Komar N, et al Experimental infection of horses with West Nile virus. Emerg Infect Dis. 2002;8: 380–386. 10.3201/eid0804.010239 11971771 PMC3393377

[8] Centers for Disease Control and Prevention (CDC). CDC. Clinical Evaluation & Disease. Last updated: June 7, 2013. Available: http://www.cdc.gov/westnile/healthCareProviders/healthCareProviders-ClinLabEval.html.

[9] Smithburn KC, Hughes TP, Burke AW, Paul JH. A Neurotropic Virus Isolated from the Blood of a Native of Uganda. Am J Trop Med Hyg. 1940; 471–92.

[10] Gubler DJ. The Continuing Spread of West Nile Virus in the Western Hemisphere. Clin Infect Dis. 2007;45: 1039–1046. 10.1086/521911 17879923

[11] Murgue B, Murri S, Triki H, Deubel V, Zeller HG. West Nile in the Mediterranean basin: 1950–2000. Ann N Y Acad Sci. 2001;951: 117–126. 11797769 10.1111/j.1749-6632.2001.tb02690.x

[12] Murgue B, Zeller H, Deubel V. The ecology and epidemiology of West Nile virus in Africa, Europe and Asia. Curr Top Microbiol Immunol. 2002;267: 195–221. 12082990 10.1007/978-3-642-59403-8_10

[13] Joubert L, Oudar J, Hannoun C, Beytout D, Corniou B, Guillon JC, et al [Epidemiology of the West Nile virus: study of a focus in Camargue. IV. Meningo-encephalomyelitis of the horse]. Ann Inst Pasteur. 1970;118: 239–247. 5461277

[14] Jupp PG. The ecology of West Nile virus in South Africa and the occurrence of outbreaks in humans. Ann N Y Acad Sci. 2001;951: 143–152. 11797772 10.1111/j.1749-6632.2001.tb02692.x

[15] Tsai TF, Popovici F, Cernescu C, Campbell GL, Nedelcu NI. West Nile encephalitis epidemic in southeastern Romania. Lancet Lond Engl. 1998;352: 767–771. 10.1016/s0140-6736(98)03538-7 9737281

[16] Platonov AE, Shipulin GA, Shipulina OY, Tyutyunnik EN, Frolochkina TI, Lanciotti RS, et al Outbreak of West Nile virus infection, Volgograd Region, Russia, 1999. Emerg Infect Dis. 2001;7: 128–132. 10.3201/eid0701.700128 11266303 PMC2631674

[17] Murgue B, Murri S, Zientara S, Durand B, Durand JP, Zeller H. West Nile outbreak in horses in southern France, 2000: the return after 35 years. Emerg Infect Dis. 2001;7: 692–696. 10.3201/eid0704.010417 11585534 PMC2631744

[18] Calistri P, Giovannini A, Hubalek Z, Ionescu A, Monaco F, Savini G, et al Epidemiology of west nile in europe and in the mediterranean basin. Open Virol J. 2010;4: 29–37. 10.2174/1874357901004020029 20517490 PMC2878979

[19] Le Guenno B, Bougermouh A, Azzam T, Bouakaz R. West Nile: a deadly virus? Lancet Lond Engl. 1996;348: 1315. 10.1016/s0140-6736(05)65799-6 8909403

[20] Tber Abdelhaq A. West Nile fever in horses in Morocco. Bull OIE. 1996; 867–869.

[21] Triki H, Murri S, Le Guenno B, Bahri O, Hili K, Sidhom M, et al [West Nile viral meningo-encephalitis in Tunisia]. Médecine Trop Rev Corps Santé Colon. 2001;61: 487–490. 11980397

[22] Malkinson M, Banet C, Weisman Y, Pokamunski S, King R, Drouet M-T, et al Introduction of West Nile virus in the Middle East by migrating white storks. Emerg Infect Dis. 2002;8: 392–397. 10.3201/eid0804.010217 11971773 PMC2730252

[23] Papa A, Danis K, Baka A, Bakas A, Dougas G, Lytras T, et al Ongoing outbreak of West Nile virus infections in humans in Greece, July-August 2010. Euro Surveill Bull Eur Sur Mal Transm Eur Commun Dis Bull. 2010;15. 10.2807/ese.15.34.19644-en 20807489

[24] The World Organisation for Animal Health (OIE). World Animal Health Information Database (WAHID) Interface [Internet]. Available: http://www.oie.int/wahis_2/public/wahid.php/Wahidhome/Home

[25] Kalaycioglu H, Korukluoglu G, Ozkul A, Oncul O, Tosun S, Karabay O, et al Emergence of West Nile virus infections in humans in Turkey, 2010 to 2011. Euro Surveill Bull Eur Sur Mal Transm Eur Commun Dis Bull. 2012;17. 22687827

[26] Sambri V, Capobianchi M, Charrel R, Fyodorova M, Gaibani P, Gould E, et al West Nile virus in Europe: emergence, epidemiology, diagnosis, treatment, and prevention. Clin Microbiol Infect Off Publ Eur Soc Clin Microbiol Infect Dis. 2013;19: 699–704. 10.1111/1469-0691.12211 23594175

[27] European Center for Disease Prevention and Control (ECDC). Historical data [Internet]. 2014. Available: http://ecdc.europa.eu/en/healthtopics/west_nile_fever/west-nile-fever-maps/pages/historical-data.aspx

[28] Giese C, Aït El Belghiti F, Barboza P, Dente M, Fabiani M, Declich S, et al West Nile virus circulation in the EpiSouth countries and neighbouring areas. Seasons 2010, 2011 and 2012 [Internet]. Available: http://www.episouthnetwork.org/sites/default/files/outputs/note_west_nile_episouth_2010_2011_2012__june2013.pdf

[29] Rizzo C, Vescio F, Declich S, Finarelli AC, Macini P, Mattivi A, et al West Nile virus transmission with human cases in Italy, August—September 2009. Euro Surveill Bull Eur Sur Mal Transm Eur Commun Dis Bull. 2009;14. 19822123

[30] Rizzo C, Salcuni P, Nicoletti L, Ciufolini MG, Russo F, Masala R, et al Epidemiological surveillance of West Nile neuroinvasive diseases in Italy, 2008 to 2011. Euro Surveill Bull Eur Sur Mal Transm Eur Commun Dis Bull. 2012;17. 22642945

[31] Hurlbut HS, Rizk F, Taylor RM, Work TH. A study of the ecology of West Nile virus in Egypt. Am J Trop Med Hyg. 1956;5: 579–620. 13354882 10.4269/ajtmh.1956.5.579

[32] Chevalier V, Tran A, Durand B. Predictive modeling of West Nile virus transmission risk in the Mediterranean Basin: how far from landing? Int J Environ Res Public Health. 2014;11: 67–90. 10.3390/ijerph110100067 PMC3924437 24362544

[33] Kilpatrick AM, Meola MA, Moudy RM, Kramer LD. Temperature, viral genetics, and the transmission of West Nile virus by *Culex pipiens* mosquitoes. PLoS Pathog. 2008;4: e1000092 10.1371/journal.ppat.1000092 18584026 PMC2430533

[34] Cornel AJ, Jupp PG, Blackburn NK. Environmental temperature on the vector competence of *Culex univittatus* (Diptera: Culicidae) for West Nile virus. J Med Entomol. 1993;30: 449–456. 8459423 10.1093/jmedent/30.2.449

[35] Richards SL, Mores CN, Lord CC, Tabachnick WJ. Impact of extrinsic incubation temperature and virus exposure on vector competence of *Culex pipiens quinquefasciatus* Say (Diptera: Culicidae) for West Nile virus. Vector Borne Zoonotic Dis Larchmt N. 2007;7: 629–636. 10.1089/vbz.2007.0101 PMC2724990 18021028

[36] Dohm DJ, Turell MJ. Effect of incubation at overwintering temperatures on the replication of West Nile Virus in New York *Culex pipiens* (Diptera: Culicidae). J Med Entomol. 2001;38: 462–464. 11372976 10.1603/0022-2585-38.3.462

[37] Paz S, Malkinson D, Green MS, Tsioni G, Papa A, Danis K, et al Permissive summer temperatures of the 2010 European West Nile fever upsurge. PloS One. 2013;8: e56398 10.1371/journal.pone.0056398 23431374 PMC3576399

[38] Tran A, Sudre B, Paz S, Rossi M, Desbrosse A, Chevalier V, et al Environmental predictors of West Nile fever risk in Europe. Int J Health Geogr. 2014;13: 26 10.1186/1476-072X-13-26 24986363 PMC4118316

[39] Hahn MB, Monaghan AJ, Hayden MH, Eisen RJ, Delorey MJ, Lindsey NP, et al Meteorological conditions associated with increased incidence of West Nile virus disease in the United States, 2004–2012. Am J Trop Med Hyg. 2015;92: 1013–1022. 10.4269/ajtmh.14-0737 25802435 PMC4426558

[40] Reisen WK, Fang Y, Martinez VM. Effects of temperature on the transmission of west nile virus by *Culex tarsalis* (Diptera: Culicidae). J Med Entomol. 2006;43: 309–317. 10.1603/0022-2585(2006)043[0309:EOTOTT]2.0.CO;2 16619616

[41] Calistri P, Ippoliti C, Candeloro L, Benjelloun A, Harrak M El, Bouchra B, et al Analysis of climatic and environmental variables associated with the occurrence of West Nile virus in Morocco. Prev Vet Med. 2013;110: 549–553. 10.1016/j.prevetmed.2013.02.011 23453893

[42] Soverow JE, Wellenius GA, Fisman DN, Mittleman MA. Infectious disease in a warming world: how weather influenced West Nile virus in the United States (2001–2005). Environ Health Perspect. 2009;117: 1049–1052. 10.1289/ehp.0800487 19654911 PMC2717128

[43] Shaman J, Day JF, Stieglitz M. Drought-induced amplification and epidemic transmission of West Nile virus in southern Florida. J Med Entomol. 2005;42: 134–141. 15799522 10.1093/jmedent/42.2.134

[44] Paz S, Semenza JC. Environmental drivers of West Nile fever epidemiology in Europe and Western Asia—a review. Int J Environ Res Public Health. 2013;10: 3543–3562. 10.3390/ijerph10083543 23939389 PMC3774453

[45] Schuster G, Ebert EE, Stevenson MA, Corner RJ, Johansen CA. Application of satellite precipitation data to analyse and model arbovirus activity in the tropics. Int J Health Geogr. 2011;10: 8 10.1186/1476-072X-10-8 21255449 PMC3038884

[46] Ward MP. Equine West Nile virus disease occurrence and the Normalized Difference Vegetation Index. Prev Vet Med. 2009;88: 205–212. 10.1016/j.prevetmed.2008.10.003 19054585

[47] Ozdenerol E, Taff GN, Akkus C. Exploring the spatio-temporal dynamics of reservoir hosts, vectors, and human hosts of West Nile virus: a review of the recent literature. Int J Environ Res Public Health. 2013;10: 5399–5432. 10.3390/ijerph10115399 24284356 PMC3863852

[48] Ward MP, Ramsay BH, Gallo K. Rural cases of equine West Nile virus encephalomyelitis and the normalized difference vegetation index. Vector Borne Zoonotic Dis Larchmt N. 2005;5: 181–188. 10.1089/vbz.2005.5.181 16011435

[49] Valiakos G, Papaspyropoulos K, Giannakopoulos A, Birtsas P, Tsiodras S, Hutchings MR, et al Use of wild bird surveillance, human case data and GIS spatial analysis for predicting spatial distributions of West Nile virus in Greece. PloS One. 2014;9: e96935 10.1371/journal.pone.0096935 24806216 PMC4013071

[50] Sugumaran R, Larson SR, Degroote JP. Spatio-temporal cluster analysis of county-based human West Nile virus incidence in the continental United States. Int J Health Geogr. 2009;8: 43 10.1186/1476-072X-8-43 19594928 PMC2717929

[51] Allan BF, Langerhans RB, Ryberg WA, Landesman WJ, Griffin NW, Katz RS, et al Ecological correlates of risk and incidence of West Nile virus in the United States. Oecologia. 2009;158: 699–708. 10.1007/s00442-008-1169-9 18941794

[52] Brownstein JS, Holford TR, Fish D. Enhancing West Nile virus surveillance, United States. Emerg Infect Dis. 2004;10: 1129–1133. 10.3201/eid1006.030457 15207069 PMC3323153

[53] Marcantonio M, Rizzoli A, Metz M, Rosà R, Marini G, Chadwick E, et al Identifying the environmental conditions favouring West Nile Virus outbreaks in Europe. PloS One. 2015;10: e0121158 10.1371/journal.pone.0121158 25803814 PMC4372576

[54] Mahalanobis PC. On the Generalized Distance in Statistics. Proc India Natl Inst Sci. 1936;2: 49–55.

[55] Rubel F, Brugger K, Hantel M, Chvala-Mannsberger S, Bakonyi T, Weissenböck H, et al Explaining Usutu virus dynamics in Austria: model development and calibration. Prev Vet Med. 2008;85: 166–186. 10.1016/j.prevetmed.2008.01.006 18314208

[56] Beck-Johnson LM, Nelson WA, Paaijmans KP, Read AF, Thomas MB, Bjørnstad ON. The effect of temperature on *Anopheles* mosquito population dynamics and the potential for malaria transmission. PloS One. 2013;8: e79276 10.1371/journal.pone.0079276 24244467 PMC3828393

[57] Laperriere V, Brugger K, Rubel F. Simulation of the seasonal cycles of bird, equine and human West Nile virus cases. Prev Vet Med. 2011;98: 99–110. 10.1016/j.prevetmed.2010.10.013 21093946

[58] Greiner M, Pfeiffer D, Smith RD. Principles and practical application of the receiver-operating characteristic analysis for diagnostic tests. Prev Vet Med. 2000;45: 23–41. 10802332 10.1016/s0167-5877(00)00115-x

[59] Swets JA. Measuring the accuracy of diagnostic systems. Science. 1988;240: 1285–1293. 3287615 10.1126/science.3287615

[60] Reeves W, Brookman B, Hammon Wm. Studies on the flight range of certain *Culex* mosquitoes, using a fluorescent-dye marker, with notes on Culiseta and Anopheles. Mosq News. 1948;8: 61–69.

[61] Berthold P, Manenti C. La migrazione degli uccelli Torino: Bollati Boringhieri; 2003.

[62] Soliman A, Mohareb E, Salman D, Saad M, Salama S, Fayez C, et al Studies on West Nile virus infection in Egypt. J Infect Public Health. 2010;3: 54–59. 10.1016/j.jiph.2009.11.002 20701892

[63] Ben Hassine T, Conte A, Calistri P, Candeloro L, Ippoliti C, De Massis F, et al Identification of Suitable Areas for West Nile Virus Circulation in Tunisia. Transbound Emerg Dis. 2015; 10.1111/tbed.12384 26032967

[64] Goldblum N, Jasinska-Klingberg W, Klingberg MA, Marberg K, Sterk VV. The natural history of West Nile Fever. I. Clinical observations during an epidemic in Israel. Am J Hyg. 1956;64: 259–269. 13372519 10.1093/oxfordjournals.aje.a119838

[65] Lanciotti RS, Roehrig JT, Deubel V, Smith J, Parker M, Steele K, et al Origin of the West Nile virus responsible for an outbreak of encephalitis in the northeastern United States. Science. 1999;286: 2333–2337. 10600742 10.1126/science.286.5448.2333

[66] Giladi M, Metzkor-Cotter E, Martin DA, Siegman-Igra Y, Korczyn AD, Rosso R, et al West Nile encephalitis in Israel, 1999: the New York connection. Emerg Infect Dis. 2001;7: 659–661. 10.3201/eid0704.010410 11585528 PMC2631756

[67] Anis E, Grotto I, Mendelson E, Bin H, Orshan L, Gandacu D, et al West Nile fever in Israel: the reemergence of an endemic disease. J Infect. 2014;68: 170–175. 10.1016/j.jinf.2013.10.009 24183889

[68] Popovici F, Sarbu A, Nicolae O, Pistol A, Cucuiu R, Stolica B, et al West Nile fever in a patient in Romania, August 2008: case report. Euro Surveill Bull Eur Sur Mal Transm Eur Commun Dis Bull. 2008;13. 18822244

[69] ECDC. European Centre for Disease Prevention and Control/WHO Regional Office for Europe West Nile virus infection outbreak in humans in Romania, 2010 ECDC; 2011.

[70] Sirbu A, Ceianu CS, Panculescu-Gatej RI, Vazquez A, Tenorio A, Rebreanu R, et al Outbreak of West Nile virus infection in humans, Romania, July to October 2010. Euro Surveill Bull Eur Sur Mal Transm Eur Commun Dis Bull. 2011;16. 21251489

[71] Di Sabatino D, Bruno R, Sauro F, Danzetta ML, Cito F, Iannetti S, et al Epidemiology of West Nile disease in Europe and in the Mediterranean Basin from 2009 to 2013. BioMed Res Int. 2014;2014: 907852 10.1155/2014/907852 25302311 PMC4180897

[72] Platonov AE, Tolpin VA, Gridneva KA, Titkov AV, Platonova OV, Kolyasnikova NM, et al The incidence of West Nile disease in Russia in relation to climatic and environmental factors. Int J Environ Res Public Health. 2014;11: 1211–1232. 10.3390/ijerph110201211 24464233 PMC3945534

[73] Hannoun C, Panthier R, Mouchet J, Eouzan JP. [ISOLATION IN FRANCE OF THE WEST NILE VIRUS FROM PATIENTS AND FROM THE VECTOR CULEX MODESTUS FICALBI]. Comptes Rendus Hebd Séances Académie Sci. 1964;259: 4170–4172. 14260659

[74] Hannoun C, Panthier R, Corniou B. Epidemiology of West Nile infections in the South of France. Bardo V, editor.Bratislava: SAS; 1969 pp. 379–387.

[75] Rollin P, Rollin D, Martin P, Baylet R, Rodhain F, Hannoun C. Résultats d’enquetes séroépidemiologiques récentes sur les arboviroses en Camargue: populations humaines, équines, bovines et aviaires. Médecine Mal Infect. 1982;12: 77–80.

[76] Filipe AR, de Andrade HR. Arboviruses in the Iberian Peninsula. Acta Virol. 1990;34: 582–591. 1983187

[77] García-Bocanegra I, Jaén-Téllez JA, Napp S, Arenas-Montes A, Fernández-Morente M, Fernández-Molera V, et al West Nile fever outbreak in horses and humans, Spain, 2010. Emerg Infect Dis. 2011;17: 2397–2399. 10.3201/eid1712.110651 22172565 PMC3311180

[78] García-Bocanegra I, Jaén-Téllez JA, Napp S, Arenas-Montes A, Fernández-Morente M, Fernández-Molera V, et al Monitoring of the West Nile virus epidemic in Spain between 2010 and 2011. Transbound Emerg Dis. 2012;59: 448–455. 10.1111/j.1865-1682.2011.01298.x 22212118

[79] Ozkul A, Ergunay K, Koysuren A, Alkan F, Arsava EM, Tezcan S, et al Concurrent occurrence of human and equine West Nile virus infections in Central Anatolia, Turkey: the first evidence for circulation of lineage 1 viruses. Int J Infect Dis IJID Off Publ Int Soc Infect Dis. 2013;17: e546–551. 10.1016/j.ijid.2013.02.005 23517780

[80] Ocal M, Onder H, Arsava EM, Alp S, Ozkul A, Ergünay K. [A case of central nervous system infection due to west nile virus lineage-1 in Ankara province, Turkey]. Mikrobiyoloji Bül. 2013;47: 164–172. 10.5578/mb.4474 23390915

[81] Ergunay K, Gunay F, Erisoz Kasap O, Oter K, Gargari S, Karaoglu T, et al Serological, molecular and entomological surveillance demonstrates widespread circulation of West Nile virus in Turkey. PLoS Negl Trop Dis. 2014;8: e3028 10.1371/journal.pntd.0003028 25058465 PMC4109882

[82] Valiakos G, Touloudi A, Athanasiou LV, Giannakopoulos A, Iacovakis C, Birtsas P, et al Serological and molecular investigation into the role of wild birds in the epidemiology of West Nile virus in Greece. Virol J. 2012;9: 266 10.1186/1743-422X-9-266 23140247 PMC3546012

[83] Komar N. West Nile viral encephalitis. Rev Sci Tech Int Off Epizoot. 2000;19: 166–176. 10.20506/rst.19.1.1201 11189714

[84] Hubálek Z, Halouzka J. West Nile Fever—a Reemerging Mosquito-Borne Viral Disease in Europe. Emerg Infect Dis. 1999;5: 643–650. 10511520 10.3201/eid0505.990505 PMC2627720

[85] Cito F, Narcisi V, Danzetta ML, Iannetti S, Sabatino DD, Bruno R, et al Analysis of surveillance systems in place in European Mediterranean countries for West Nile virus (WNV) and Rift Valley fever (RVF). Transbound Emerg Dis. 2013;60 Suppl 2: 40–44. 10.1111/tbed.12124 24589100

[86] Childs JE, Gordon ER. Surveillance and control of zoonotic agents prior to disease detection in humans. Mt Sinai J Med N Y. 2009;76: 421–428. 10.1002/msj.20133 19787654

