# Supplementary figures and images for: Spatio-Temporal Identification of Areas Suitable for West Nile Disease in the Mediterranean Basin and Central Europe
Source: PLoS One. 2015 Dec 30;10(12):e0146024. doi: 10.1371/journal.pone.0146024 (PMC4696814; doi:10.1371/journal.pone.0146024)

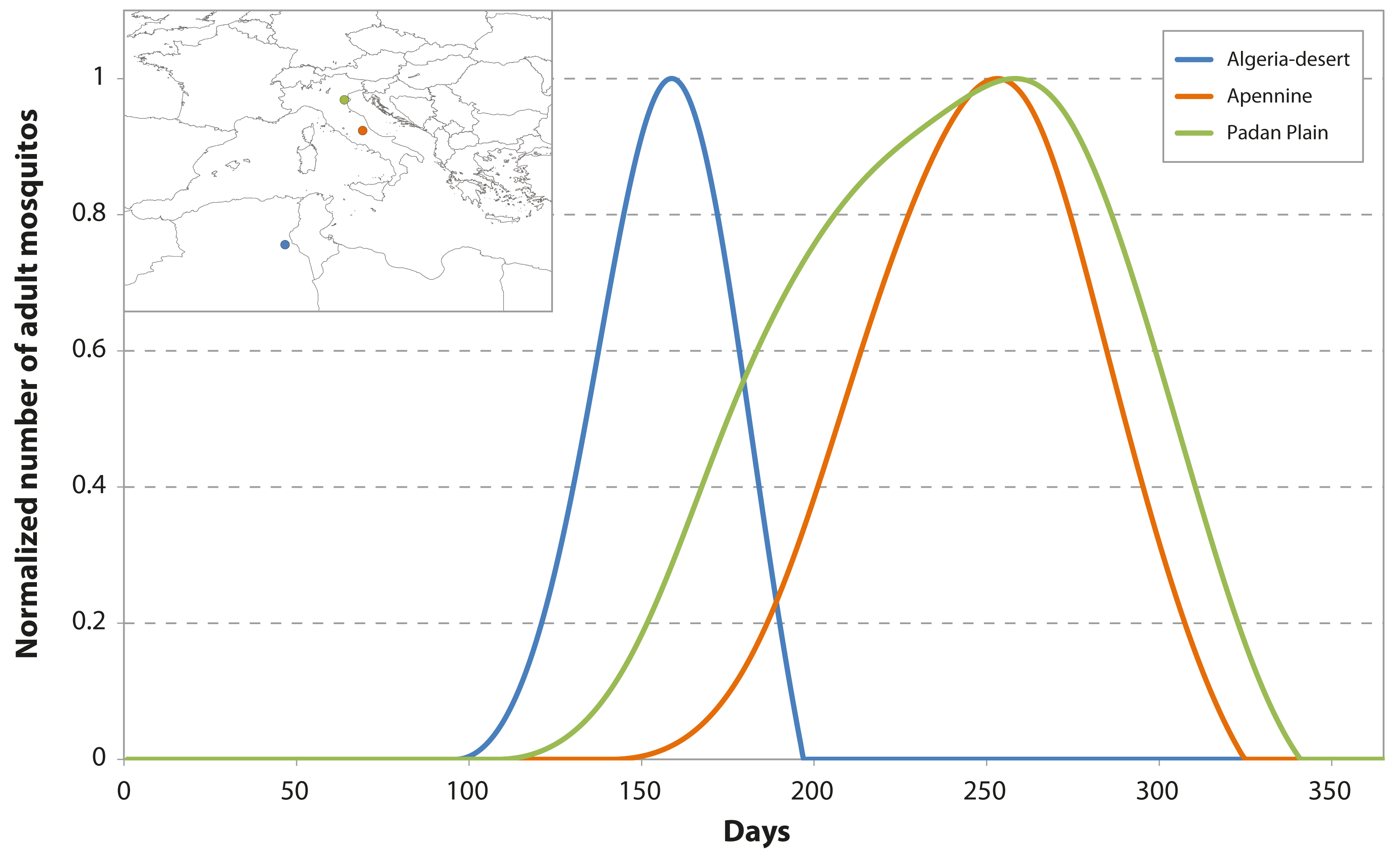

Supplement: S2 Fig — (JPG) [file pone.0146024.s002.jpg]
