# Supplementary material for: Spatio-Temporal Identification of Areas Suitable for West Nile Disease in the Mediterranean Basin and Central Europe
Source: PLoS One. 2015 Dec 30;10(12):e0146024. doi: 10.1371/journal.pone.0146024 (PMC4696814; doi:10.1371/journal.pone.0146024)
Supplement: S2 Table — (DOCX) [file pone.0146024.s004.docx]

**S2 Table. Formulas, parameters and references of the mosquito’s growth model: birth and mortality parameters and initial values.**

| **Parameter** | | **Value** | **Description** | **Reference** |
| --- | --- | --- | --- | --- |
| **BIRTH PARAMETERS** | | |  |  |
| $\boldsymbol{k}$ | $k\left( T \right)=\frac{0.344}{1+1.231 exp(-0.184 \left( T-20 \right))}$ | | logistic (S-shaped) function, considering the biting (*i.e.* contact) rate | [55] |
| $\boldsymbol{\delta}$ | $\delta\left( D \right)=1-\frac{1}{1+1775.7 exp[1.559 \left( D-18.177 \right)]}$ | | logistic function describing the fraction of active mosquitoes, *i.e.* non-diapausing mosquitoes | [55] |
| **D** | $D\left( \epsilon,\varphi\right)=7.639\arcsin\left( \tan\epsilon\tan\varphi+\frac{0.0146}{\cos\varepsilon cos\varphi} \right)+12$ | | *D* is the daytime length in hours | [55] |
| $\boldsymbol{\epsilon}$ | $\epsilon=0.409 sin\left( \frac{2\pi(d-80)}{365} \right)$ | | declination of the sun | [55] |
| ***D*** | 1, …, 365 | | day of the year |  |
| **Φ** | 45*π/180 | | Mean geographical latitude |  |
| **MORTALITY PARAMETERS** | | |  |  |
| $\boldsymbol{\gamma}_{\boldsymbol{L}}$ | | 3/2 | Estimate of the proportion of time spent in larval stage | [56] |
| $\boldsymbol{\mu}_{\boldsymbol{3}}$ | | 0.02 | Fit scalar in larvae mortality rate function | [56] |
| $\boldsymbol{\mu}_{\boldsymbol{4}}$ | | 23 | Fit scalar in larvae mortality rate function | [56] |
| $\boldsymbol{\mu}_{\boldsymbol{5}}$ | | 6.5 | Fit scalar in larvae mortality rate function | [56] |
| **INITIAL VALUES** | | |  |  |
| **L_0_** | | 0 | Larvae | [57] |
| **N_0_** | | 500,000 | Mosquitoes adults (minimum population in winter) | [57] |
